# TPM4 condensates glycolytic enzymes and facilitates actin reorganization under hyperosmotic stress

**DOI:** 10.1038/s41421-024-00744-2

**Published:** 2024-12-03

**Authors:** Wenzhong Yang, Yuan Wang, Geyao Liu, Yan Wang, Congying Wu

**Affiliations:** 1 Institute of Systems Biomedicine, School of Basic Medical Sciences, Peking University Health Science Center, Beijing, China; 2 International Cancer Institute, Peking University, Beijing, China; 3 Institute of Advanced Clinical Medicine, State Key Laboratory of Molecular Oncology, Beijing, China

**Keywords:** Actin, Stress fibres

## Abstract

Actin homeostasis is fundamental for cell structure and consumes a large portion of cellular ATP. It has been documented in the literature that certain glycolytic enzymes can interact with actin, indicating an intricate interplay between the cytoskeleton and cellular metabolism. Here we report that hyperosmotic stress triggers actin severing and subsequent phase separation of the actin-binding protein tropomyosin 4 (TPM4). TPM4 condensates recruit glycolytic enzymes such as HK2, PFKM, and PKM2, while wetting actin filaments. Notably, the condensates of TPM4 and glycolytic enzymes are enriched of NADH and ATP, suggestive of their functional importance in cell metabolism. At cellular level, actin filament assembly is enhanced upon hyperosmotic stress and TPM4 condensation, while depletion of TPM4 impairs osmolarity-induced actin reorganization. At tissue level, colocalized condensates of TPM4 and glycolytic enzymes are observed in renal tissues subjected to hyperosmotic stress. Together, our findings suggest that stress-induced actin perturbation may act on TPM4 to organize glycolytic hubs that tether energy production to cytoskeletal reorganization.

## Introduction

Actin, the most abundant protein in eukaryotic cells, polymerizes through nucleation and elongation from globular actin monomers (G-actin) to form filamentous actin (F-actin). The dynamic rearrangement of the actin cytoskeleton is a highly energy-consuming process. Early studies found that the turnover of F-actin accounted for 50% of total ATP consumption in unstimulated platelets and neurons^1,2^. The direct interactions between glycolytic enzymes and actin indicate that glycolysis may provide the necessary fuel for actin rearrangement^3–5^. Actin cytoskeleton has recently been shown to adjust glycolytic level. Soft substrate induces stress fiber disassembly, releasing E3 ubiquitin ligase tripartite motif (TRIM)-containing protein 21 (TRIM21) to degrade phosphofructokinase (PFK) and thus downregulate glycolysis^6^. Insulin stimulation triggers F-actin remodeling in epithelial cells through the PI3K-PIP3-Rac1 pathway, increasing the amount of actin-free aldolase (ALDOA) in the cytoplasm, thereby enhancing glycolytic flux^7^. Purified PFK protein has long been documented to bind polymerized actin and form paracrystal in vitro^3^. In yeast, F-actin acts as a scaffold for glycolytic enzymes to form a multi-enzymatic glycolytic complex^8^. It has been hypothesized that actin cytoskeleton can compartmentalize glycolytic enzymes^9^. Functions of the interplays between F-actin and these enzymes including direct interactions and indirect regulation through complex signaling pathways and protein modifications have just started to be elucidated.

Various cellular stress conditions, including hyperosmotic stress, impact cell morphology and metabolism. In turn, cells rewire their cytoskeleton and energy production to survive harsh conditions. During starvation, cells adapt by inhibiting growth, inducing autophagy, and reprogramming metabolism^10^ to optimize limited energy supplies, where actin cytoskeleton reorganizes to assist with autophagosome formation^11^ and cargo trafficking^12^ as well as cell death^13^. Changes in extracellular osmolarity not only influence the life cycle of single cells, but also challenge multicellular organisms by perturbing the cell cortex and trafficking systems, cytoplasm viscosity, nuclear volume and protein stability^14–16^. Notably, hyperosmotic stress induces the condensation of multivalent proteins^17,18^, potentially influencing cellular adaptation. Whether and how phase separation is involved in actin cytoskeletal remodeling under hyperosmotic stress remains unclear.

Actin dynamics are critical for various cellular processes, including signal transduction pathway, cell shape maintenance, and motility. Recent studies have highlighted the role of biomolecular condensates formed through liquid-liquid phase separation (LLPS) in regulating actin dynamics. For instance, the multivalent interactions among Nephrin, Nck, and N-WASP proteins facilitate their clustering on lipid bilayers, leading to phase separation into micrometer-sized structures that enhance Arp2/3 complex-dependent actin assembly^19^. Additionally, TCR activation leads to phase separation of kinases and recruitment of actin regulators, thereby enhancing actin filament assembly by recruiting and organizing actin regulators^20^. Actin itself can undergo phase separation in the presence of cross-linkers such as filamin, which condense short actin filaments into dynamic structures, underscoring the interplay between actin organization and LLPS ^21^. Moreover, biomolecular condensates can modulate the activity of actin regulatory proteins. For example, the LLPS of Nephrin–Nck–N-WASP on lipid bilayers increases the membrane dwelling time of N-WASP and the Arp2/3 complex, thereby enhancing actin assembly^22^. Such mechanism is particularly evident in excitatory postsynaptic density (PSD) structures, where condensates facilitate actin bundle formation and regulate synaptic function through dynamic phase separation ^23^. Notably, in vitro reconstituted PSD condensates can promote actin polymerization and F-actin bundling independent of actin regulatory proteins^24^. Additionally, condensates containing Nck and WASP/N-WASP bind to actin filaments and move with them, while condensates lacking these proteins do not bind efficiently to actin and move differently ^25^. The intricate interplay between phase separation and actin dynamics remains an exciting area for research, promising deeper understanding of cellular functions and disease mechanisms.

LLPS serves as a reaction crucible for biochemical reactions and recruits specific interacting factors, thereby promoting reaction progress^26,27^. Multienzyme metabolic assemblies have been identified in various tissues and species. For instance, “glucosome” is a reversibly organized assembly of enzymes involved in both glycolysis and gluconeogenesis, regulating glucose flux at the subcellular level in human cancer cells. Glucosomes contain enzymes such as PFK, fructose-1,6-bisphosphatase (FBPase), phosphoenolpyruvate carboxykinase (PEPCK), and pyruvate kinase M (PKM). Small-sized glucosomes primarily promote glycolysis, while medium- and large-sized ones preferentially partition glucose flux into the pentose phosphate pathway (PPP) and serine biosynthesis, respectively^28^. During G1 phase, increased small and medium glucosomes in Hs578T cells suggest heightened glycolysis and PPP, while decreased small glucosomes in G2/M-arrested cells indicate reduced glycolysis in G2 phase^29^. Moreover, under hypoxic stress, yeast and *Caenorhabditis elegans* neurons form “G bodies” or “glycolytic granules” containing PFK and other glycolytic enzymes. These structures are similar to stress granules and P bodies formed through phase separation^30–35^. The formation of these structures may enhance the activity of the glycolytic pathway, optimize reaction rates, and contribute to ATP production, forming a functional unit known as “metabolon”^30–32,36^. Mutants unable to form these structures exhibit decreased cellular viability or impaired synaptic function ^31,32^. Nonetheless, a systematic mechanism for actin cytoskeleton, phase separation and glycolysis remained to be established.

Here we discovered that actin-binding protein tropomyosin 4 (TPM4) can undergo phase separation, forming condensates that wet actin filaments. These TPM4 condensates play a functional role in recruiting crucial glycolytic enzymes such as hexokinase 2 (HK2), phosphofructokinase muscle type (PFKM), and pyruvate kinase M2 (PKM2), promoting glycolysis. Our findings suggest that TPM4 condensates act as a central hub for osmolarity-induced glycolysis upregulation, providing a novel model that links actin cytoskeleton and glycolysis.

## Results

### Dense regions of actin-binding protein contain glycolytic enzymes upon hyperosmotic stress

**Fig. 1:**
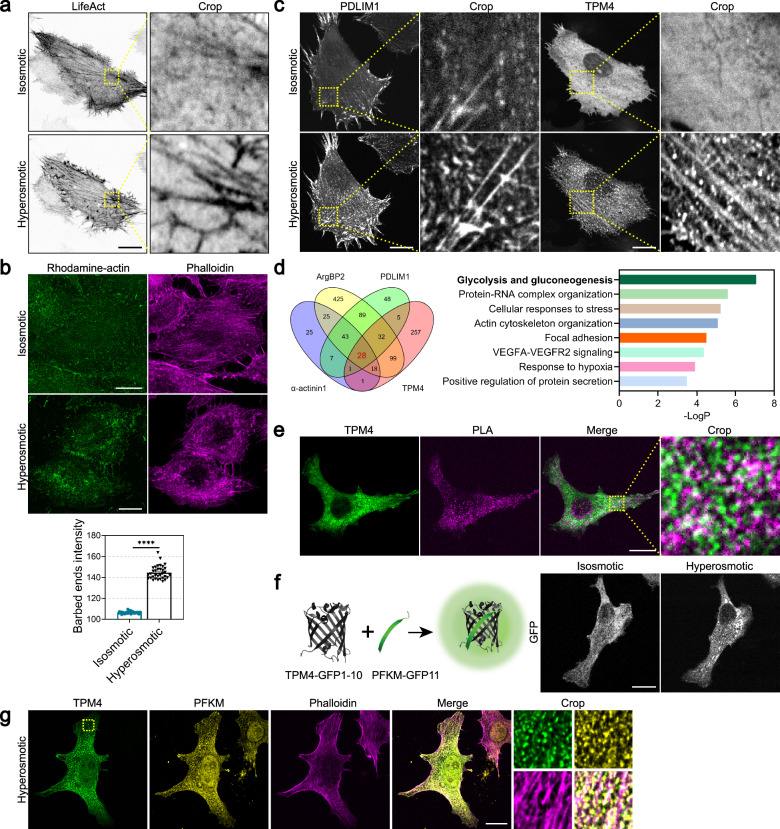
Dense regions of actin-binding protein contain glycolytic enzymes upon hyperosmotic stress. **a** Representative images of MDA-MB-231 cells expressing LifeAct-EGFP before (isosmotic) and after (hyperosmotic) 100 mM sorbitol treatment for 1 h. Scale bar, 20 μm. **b** Top: barbed end assay showing the distribution of rhodamine-actin in MDA-MB-231 cells before (isosmotic) and after (hyperosmotic) 100 mM sorbitol treatment for 3 min. Actin filaments are labeled by phalloidin. Scale bar, 20 μm. Bottom: quantification of barbed end intensity in MDA-MB-231 cells before (isosmotic) and after (hyperosmotic) 100 mM sorbitol treatment for 3 min. *n* = 34; error bar: mean with SEM; ****P < 0.0001 by unpaired *t*-test. **c** Representative images of MDA-MB-231 cells expressing PDLIM1-AcGFP or TPM4-AcGFP before (isosmotic) and after (hyperosmotic) 100 mM sorbitol treatment for 3 min. Scale bars, 20 μm. **d** Left: Venn diagram illustrating the identified proteins by TurboID proximity labeling/mass spectrometry using ArgBP2, PDLIM1, α-actinin1 and TPM4 fused with TurboID. Right: enrichment analysis by Metascape showing top enriched signaling pathways. **e** PLA assay showing the interaction between TPM4-AcGFP and PFKM in MDA-MB-231 cells under hyperosmotic condition (100 mM sorbitol for 3 min). Scale bar, 20 μm. **f** Left: schematic diagram of split-GFP system expressing TPM4-GFP1–10 and PFKM-GFP11. Right: representative images of MDA-MB-231 cells expressing split-GFP constructs before (isosmotic) and after (hyperosmotic) 100 mM sorbitol treatment for 3 min. Scale bar, 20 μm. **g** Representative images of PFKM (yellow) and phalloidin (magenta) immunofluorescence staining in MDA-MB-231 cells expressing TPM4-AcGFP under hyperosmotic condition (100 mM sorbitol for 3 min). Scale bar, 20 μm.

To visualize actin cytoskeleton in response to hyperosmotic stress, we expressed LifeAct-EGFP to label actin filaments while adding sorbitol to the cell culture medium for the increase of osmolarity. Live-cell imaging revealed that cell body shrank, followed by dynamic actin network reorganization and thickening of individual stress fibers, indicative of hierarchical and coordinated biochemical and biomechanical cellular response to hyperosmotic stress (Fig. 1a). Actin assembles actively at the barbed ends. We adapted a barbed end assay ^37^ to probe de novo actin polymerization sites. To localize free barbed ends, cells were permeabilized in detergent containing rhodamine-actin at concentrations sufficient to support only barbed end polymerization ^38^. We observed that cells exhibited increased barbed ends under hyperosmotic stress (Fig. 1b), indicating that hyperosmotic stress may induce actin filament breakage and generation of free barbed ends.

These observations led to the intriguing question: how do cells regulate actin reorganization under hyperosmotic stress? Actin-binding proteins are known to regulate actin dynamics, organization, and stability by controlling monomeric availability, nucleation, elongation, and depolymerization^39^. Interestingly, we observed increased localization of various actin-binding proteins such as TPM4, PDLIM1, α-actinin1, and ArgBP2 on actin filaments in response to hyperosmotic stress (Fig. 1c; Supplementary Fig. S1a). These findings suggest that the remodeling process of actin filaments under hyperosmotic stress may involve the recruitment of multiple components. To investigate the recruited components and their functions in this process, we performed TurboID proximity labeling^40^/mass spectrometry using four major actin-binding proteins (α-actinin1, PDLIM1, ArgBP2, and TPM4), which have been reported to form dense bodies^41–43^ on actin filaments or function as side-binding proteins along actin filaments^44^. Analyzing the results of the four identified groups of mass spectrometry, we found 28 proteins shared by all four groups (Fig. 1d; Supplementary Fig. S1b). Subsequent enrichment analysis using the “Metascape^45^” platform revealed that the “glycolysis and gluconeogenesis” pathway ranked highest in significance (Fig. 1d), and among them resided the crucial rate-limiting enzyme PFKM involved in glycolysis.

Actin polymerization demands of high energy consumption. Glycolysis is a quick ATP-producing pathway. It has been documented that purified PFK can interact with actin filaments in vitro^3^. However, the role of actin filaments in regulating PFK in the complex cellular environment remains unclear. We then wondered whether actin-binding proteins such as TPM4 may have a secondary function in facilitating energy support for reorganizing actin cytoskeleton under hyperosmotic stress. To answer this, we first investigated whether TPM4 could interact with glycolytic enzymes. Through the proximity ligation assay (PLA) technique, we identified positive signals resulting from the interaction between TPM4 and PFKM under hyperosmotic stress (Fig. 1e). In addition, we employed split-GFP system^46^, wherein TPM4 and PFKM were respectively fused to distinct fragments of the fluorescein moiety of GFP. When shifting cells to hyperosmotic medium, we instantaneously detected enhanced formation of green fluorescent foci (Fig. 1f). Furthermore, PFKM showed strong co-localization with TPM4 under hyperosmotic stress (Pearson’s coefficient = 0.77), and these colocalized sites were closely associated with actin filaments (Fig. 1g).

Together, we detected that hyperosmotic stress promoted an upsurge in actin filament assembly, and the actin-binding protein TPM4 showed enhanced localization along actin filaments with glycolytic enzymes such as PFKM.

### TPM4 can form condensates through phase separation

**Fig. 2:**
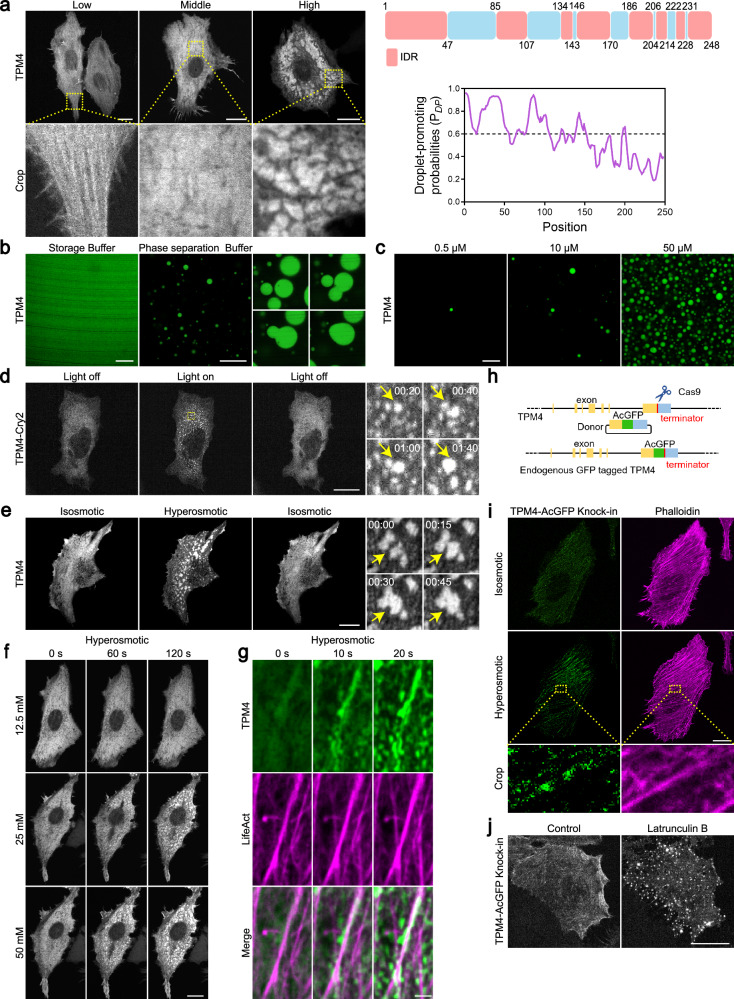
TPM4 can undergo phase separation. **a** Left: representative images of MDA-MB-231 cells expressing low, medium and high levels of TPM4-AcGFP. Scale bars, 20 μm. Top right: IDR (red) and non-IDR (blue) regions of TPM4 according to MobiDB (https://mobidb.bio.unipd.it/); bottom right: residue-based *P*_DP_ of TPM4 according to FuzDrop (https://fuzdrop.bio.unipd.it/predictor). **b** Representative images of purified TPM4-AcGFP protein in storage and phase separation buffer. Scale bars, 10 μm. Zoomed images showing the fusion of TPM4 droplets in vitro. **c** Representative images of TPM4-AcGFP droplets with different protein concentrations (0.5 μM, 10 μM, 50 μM) in phase separation buffer. Scale bar, 10 μm. **d** Left: representative images of MDA-MB-231 cells expressing TPM4-mCherry-Cry2 upon blue light exposure and withdrawal. Scale bar, 20 μm. Right: zoomed images showing the formation and fusion of TPM4 condensates upon blue light exposure within 2 min. **e** Left: representative images of MDA-MB-231 cells expressing TPM4-AcGFP under 100 mM sorbitol treatment and washout. Scale bar, 20 μm. Right: zoomed images showing the fusion events of TPM4 condensates. **f** Representative time-lapse images of MDA-MB-231 cells expressing TPM4-AcGFP treated with 12.5 mM, 25 mM, 50 mM sorbitol within 2 min. Scale bar, 20 μm. **g** Representative time-lapse images of TPM4-AcGFP and LifeAct-mScarlet in MDA-MB-231 cells treated with 100 mM sorbitol within 20 s. Scale bar, 2 μm. **h** Schematic illustration of CRISPR-Cas9-mediated endogenous tagging of *TPM4* gene with GFP at C-terminus. **i** Representative images of endogenous TPM4-AcGFP in MDA-MB-231 cells before (isosmotic) and after (hyperosmotic) 100 mM sorbitol treatment for 3 min. Scale bar, 20 μm. **j** Representative images of endogenous TPM4-AcGFP in MDA-MB-231 cells before and after 2.5 μM latrunculin B treatment for 1 h. Scale bar, 20 μm.

When we expressed TPM4-AcGFP in cells, we observed TPM4 in different morphologies — lining with the actin filaments, diffusive, or condensated in the cytoplasm (Fig. 2a, left panels), the last of which hinted a propensity to undergo phase separation. Prediction analysis revealed that ~60% region of TPM4 were intrinsically disordered regions (IDRs) (Fig. 2a, top right panel), indicating its intrinsic capacity of phase separation. Furthermore, FuzDrop^47–49^ analysis revealed that the droplet-promoting probability (*P*_DP_) of TPM4 was 0.89 (Fig. 2a, bottom right panel), and proteins with *P*_DP_ ≥ 0.60 are predicted to spontaneously phase separate under physiological conditions, thus supporting that TPM4 has intrinsic capacity for phase separation.

We then carried out in vitro phase separation assay using affinity-purified TPM4 protein (Supplementary Fig. S3b) and observed concentration-dependent droplet formation (Fig. 2b, c). These droplets underwent rapid fusion when encountering each other (Fig. 2b), suggesting a liquid state, which is typical of condensates formed via phase separation. Moreover, we employed an optogenetic approach based on the “optoDroplet” system^50^ and found reversible formation of TPM4-Cry2 droplets upon blue light exposure in multiple cell lines (Fig. 2d; Supplementary Fig. S2a). These data support the notion that TPM4 can undergo phase separation.

Recent work has shown that hyperosmotic stress could induce phase separation^17,18^. Following the results that hyperosmotic stress promoted actin remodeling, we asked whether phase separation induced by hyperosmotic stress is involved in this process. Indeed, we observed rapid formation of TPM4 condensates under hyperosmotic stress (100 mM sorbitol; 400 mOsm) (Fig. 2e). These TPM4 condensates were dynamic and reversible upon washing out of the hyperosmotic medium. TPM4 puncta were visible with sorbitol stress as low as 25 mM (325 mOsm), and the number of puncta per cell increased with higher osmolarity (Fig. 2f). Other hyperosmotic stressors exerted similar effects on TPM4 puncta formation (Supplementary Fig. S2b). Co-transfection of TPM4-AcGFP and LifeAct-mScarlet revealed that TPM4 condensates wetted actin filaments under hyperosmotic stress (Fig. 2g; Supplementary Video S1). Fusion events of TPM4 condensates occurred along actin filaments (Supplementary Fig. S2c and Video S2).

To determine whether these findings are relevant at endogenous levels of TPM4, we generated a TPM4 knock-in (KI) cell line in which AcGFP was inserted into the C-terminus (referred to as TPM4 KI hereafter, Fig. 2h and Supplementary Fig. S2d). When subjected to hyperosmotic stress, the TPM4 KI cells exhibited dramatically enhanced TPM4 localization on actin filaments (Fig. 2i). Besides, we noticed that the signals of endogenous TPM4 on actin filaments are discontinuous and condensate-like (Fig. 2i; Supplementary Fig. S2e). Interestingly, when we depolymerized the actin with latrunculin B, which would also release actin-binding TPM4, we observed more pronounced TPM4 condensates in the cytoplasm (Fig. 2j).

These results support that TPM4 can undergo phase separation, forming condensates that wet actin filaments under hyperosmotic stress.

### TPM4 phase separation can recruit glycolytic enzymes

**Fig. 3:**
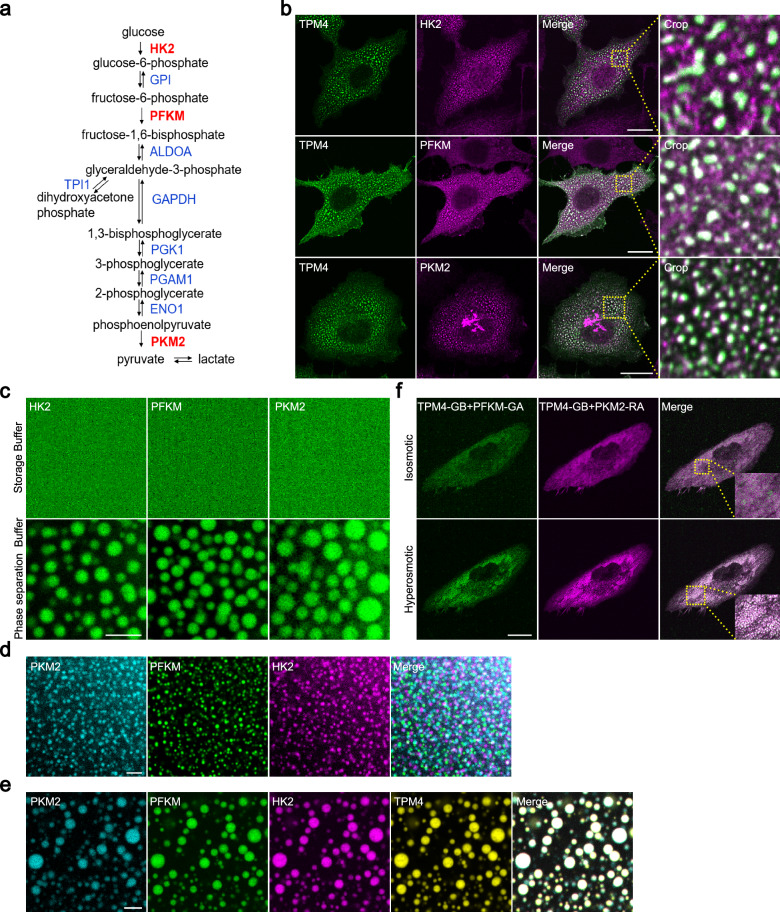
TPM4 condensates recruit rate-limiting glycolytic enzymes. **a** Flow chart showing the steps of glycolysis and key enzymes involved. Three rate-limiting enzymes are highlighted in red. **b** Representative images of HK2, PFKM and PKM2 immunofluorescence staining in MDA-MB-231 cells expressing TPM4-AcGFP under hyperosmotic condition (100 mM sorbitol for 3 min). Scale bars, 20 μm. **c** Representative images of purified HK2-iRFP, PFKM-AcGFP and PKM2-BFP in storage and phase separation buffer. Scale bar, 5 μm. **d** Representative images of purified PKM2-BFP separately with PFKM-AcGFP and HK2-iRFP proteins in phase separation buffer. Scale bar, 5 μm. **e** Representative co-phase images of purified PKM2-BFP, PFKM-AcGFP and HK2-iRFP with TPM4-RFP in phase separation buffer. Scale bar, 5 μm. **f** Representative images of MDA-MB-231 cells expressing TPM4-GB, PFKM-GA and PKM2-RA before (isosmotic) and after (hyperosmotic) 100 mM sorbitol treatment for 3 min. Scale bar, 20 μm.

Next, we set out to investigate the role of TPM4 phase separation and its potential relationship with glycolysis. Glycolysis involves at least ten steps, three of which require rate-limiting enzymes PFKM, HK2 and PKM2 (Fig. 3a). Immunofluorescence staining revealed the presence of PFKM, HK2 and PKM2 within TPM4 condensates upon hyperosmotic stress (Fig. 3b). Apart from these, we also identified endogenous signals of other glycolytic enzymes within the TPM4 condensates, including GPI (phosphoglucose isomerase), ALDOA, TPI (triosephosphate isomerase), GAPDH (glyceraldehyde-3-phosphate dehydrogenase), PGK1 (phosphoglycerokinase 1), PGAM1 (phosphoglycerate mutase 1), and ENO1 (enolase 1) (Supplementary Fig. S3a).

We purified the PFKM, HK2 and PKM2, and observed that all of them could form condensates in vitro, respectively (Fig. 3c; Supplementary Fig. S3b). However, when mixed together, PFKM, HK2 and PKM2 formed distinct condensates (Fig. 3d). Interestingly, by adding TPM4 to the 3-protein system and conducting a 4-color imaging phase separation assay, we observed that these glycolytic enzymes formed colocalized 4-color condensates (Fig. 3e). These results suggest that TPM4 may act as a scaffold to organize multiple glycolytic enzymes.

FPX (fluorescent protein exchange) could be used to image dynamic and reversible protein–protein interactions. This system involves two monomers — one (GA or RA) contains a chromophore that is quenched in the monomeric state, while the other (GB) acts only to substantially increase the fluorescence when forming heterodimer with GA or RA (GA/GB or RA/GB)^51^. To visualize the recruitment process of glycolytic enzymes by TPM4 within cells, we fused GA, GB and RA tags to PFKM, TPM4 and PKM2, respectively. Green fluorescence was detected due to the interaction between PFKM-GA and TPM4-GB (Fig. 3f, upper left) and red fluorescence was detected due to the interaction between TPM4-GB and PKM2-RA (Fig. 3f, upper middle). Notably, hyperosmotic stress induced the formation of both green and red condensates and they colocalized (Fig. 3f, bottom and right), supporting that TPM4 can recruit two types of glycolytic enzymes simultaneously.

In conclusion, these findings imply that TPM4 can enrich glycolytic enzymes through phase separation.

### TPM4 phase separation is correlated with elevated glycolysis

**Fig. 4:**
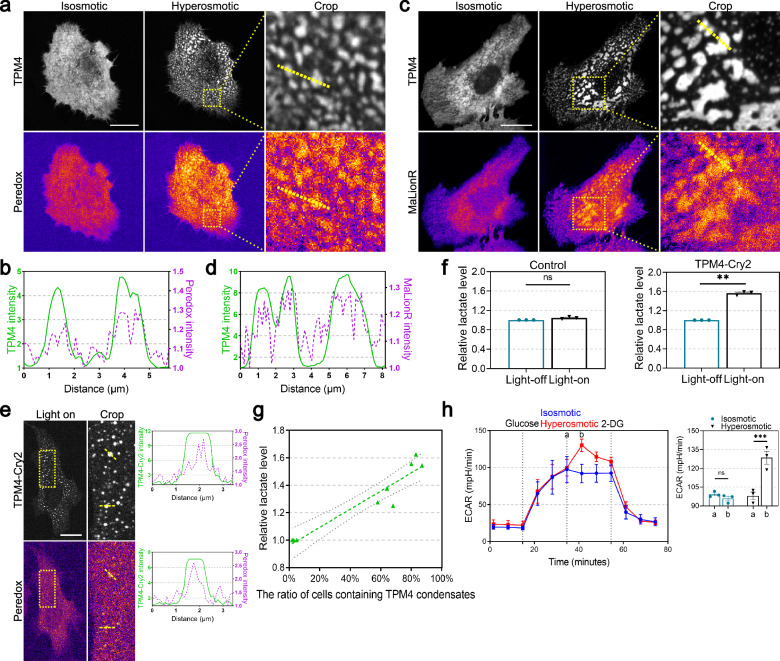
TPM4 phase separation is correlated with elevated glycolysis. **a** Representative images of TPM4 and NADH signals by Peredox probe in MDA-MB-231 cells expressing TPM4-AcGFP before (isosmotic) and after (hyperosmotic) 100 mM sorbitol treatment for 3 min. Scale bar, 20 μm. **b** Line scan plot showing the intensity distribution of TPM4 (green) and NADH signals (magenta) along the yellow line shown in **a** under hyperosmotic condition. **c** Representative images of ATP signals by MaLionR probe in MDA-MB-231 cells expressing TPM4-AcGFP before (isosmotic) and after (hyperosmotic) 100 mM sorbitol treatment for 3 min. Scale bar, 20 μm. **d** Line scan plot showing the intensity distribution of TPM4 (green) and ATP signals (magenta) along the yellow line shown in **c** under hyperosmotic condition. **e** Left: representative images of TPM4 and NADH signals in MDA-MB-231 cells expressing TPM4-mCherry-Cry2 upon blue light exposure. Dashed boxes are zoomed in on the right. Scale bar, 20 μm. Right: line scan plot showing the intensity distribution of TPM4 and NADH signals along the yellow line. **f** Quantification of relative lactate level in cells expressing non-optoDroplet-TPM4 (TPM4-AcGFP) or optoDroplet-TPM4 (TPM4-mCherry-Cry2) before and after blue light exposure for 10 min. *n* = 3 independent experiments; error bar: mean with SEM; ns = 0.0898, **P = 0.0015 by paired *t*-test. **g** Lactate production gradually rises with an increasing percentage of cells displaying TPM4 condensates. The *x*-axis shows the percentage of cells with TPM4 condensates (*n* = 100 cells per condition, repeated three times independently) across different experimental conditions, including control, 100 mM sorbitol and 250 mM sorbitol hyperosmotic treatment. The *y*-axis shows levels of lactate produced by cells under the same conditions (*n* = 3 independent experiments; error bar: mean with 95% CI). **h** Left: quantitative curves of ECAR in MDA-MB-231 cells treated with Seahorse basic medium (isosmotic) or 100 mM sorbitol (hyperosmotic) (*n* = 3 independent experiments; error bar: mean with SEM). Sorbitol was added at time point a, and ECAR reaches its highest level at time point b. Right: quantification of ECAR in isosmotic and hyperosmotic groups at time points a and b. *n* = 3 independent experiments; error bar: mean with SEM; isosmotic ns = 0.8752, hyperosmotic ***P_ab_ = 0.0007 by two-way ANOVA.

Having observed that hyperosmotic stress enhanced TPM4 phase separation and recruited glycolytic enzymes, we then asked whether these TPM4 condensates are functionally associated with glycolysis. First, to monitor the production of the glycolysis intermediate NADH in the process of glyceraldehyde 3-phosphate (G3P) conversion into 1,3-bisphosphoglyceric acid (1,3-BPG), we employed the Peredox probe, which contains a bacterial NADH-binding protein Rex and undergoes conformational change upon NADH binding. This shift from an open conformation to a closed one results in the fluorescence change of the linked fluorescent protein^52^. Upon switching into hyperosmotic medium, increased fluorescent intensity was readily observed and resided in a condensed fashion. Notably, the enhanced NADH signal colocalized with TPM4 intensity, as revealed by line scanning (Pearson’s coefficient = 0.859) (Fig. 4a, b).

Second, we sought to monitor ATP level as an indicator of net energy production in cells under hyperosmotic stress. The MaLionR probe, which inserts the ATP-binding ε subunit of the bacterial FoF1-ATP synthase into the red fluorescent protein mApple, changes fluorescence when triggered by ATP binding^53^. Using this probe, we observed that TPM4 condensates induced by hyperosmotic stress exhibited significantly higher levels of ATP compared to surrounding regions (TPM4-MaLionR, Pearson’s coefficient = 0.756) (Fig. 4c, d). These data support that TPM4 condensates induced by hyperosmotic stress can serve as hotspots for glycolysis and energy production.

Third, we asked whether opto-genetically induced TPM4 phase separation would be competent to concentrate glycolysis. We shed blue light to TPM4-Cry2-expressing cells and observed that TPM4 condensates showed significantly higher NADH levels compared to surrounding regions (Fig. 4e). Moreover, we shed blue light to TPM4-Cry2-expressing cells and detected the overall glycolytic level in cells using the L-lactate assay kit. We observed a significant increase in lactate production by cells with TPM4 phase separation. We used non-optodroplet-TPM4 expressing cells (TPM4-AcGFP) as a negative control and found no noticeable changes in lactate production after exposing these cells to the same blue light (Fig. 4f). These results support the functional association between TPM4 phase separation and elevated glycolysis.

Fourth, in support of the notion that hyperosmotic stress triggers functional TPM4-glycolytic enzyme condensation, we detected the overall glycolytic level in cells by two means, the L-lactate assay and Seahorse assay. We observed that a gradual rise in lactate production by cells was correlated with a higher ratio of cells with TPM4 condensates (Fig. 4g). When we switched normal medium to hyperosmotic medium while monitoring bulk glycolysis using the Seahorse assay, we found that hyperosmotic stress rapidly increased the extracellular acidification rate (ECAR). Subsequently, the use of 2-deoxyglucose (2-DG), a competitive inhibitor of the glycolytic rate-limiting enzyme HK2, significantly reduced ECAR (Fig. 4h). This suggests that the increase in ECAR induced by hyperosmotic stress originates from the glycolytic pathway.

Overall, these results support that TPM4 phase separation is functionally correlated with elevated glycolysis.

### TPM4 mediates actin reorganization coupled with glycolysis regulation

**Fig. 5:**
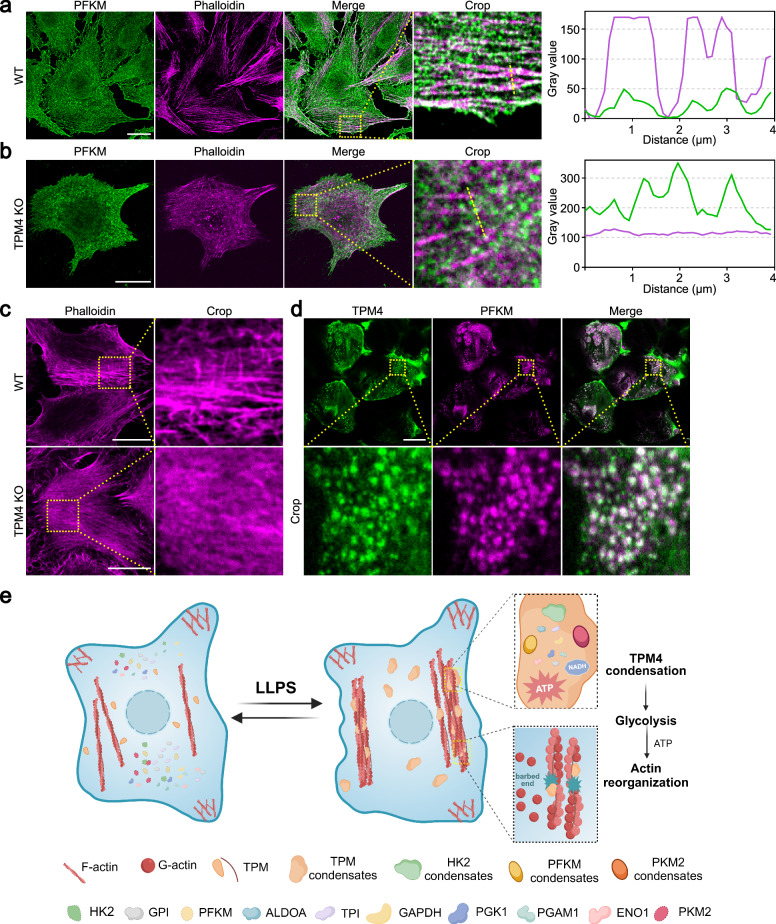
TPM4 mediates actin reorganization coupled with regulation of glycolysis. **a** Left: representative images of PFKM (green) and phalloidin (magenta) immunofluorescence staining in MDA-MB-231 wild-type cells treated with 100 mM sorbitol for 3 min. Scale bar, 20 μm. Right: line scan plot showing the intensity distribution of PFKM and actin filaments along the yellow line. **b** Representative images of PFKM (green) and phalloidin (magenta) immunofluorescence staining in MDA-MB-231 TPM4 KO cells treated with 100 mM sorbitol for 3 min. Scale bar, 20 μm. Right: line scan plot showing the intensity distribution of PFKM and actin filaments along the yellow line. **c** Representative images of phalloidin (magenta) immunofluorescence staining in wild-type and TPM4 KO cells treated with 100 mM sorbitol for 1 h. Scale bars, 20 μm. **d** Representative images of TPM4 (green) and PFKM (magenta) immunofluorescence staining in mouse kidney tissue sections. Scale bar, 20 μm. **e** A model (created with BioRender.com) for TPM4 phase separation-mediated reorganization of multiple glycolytic enzymes. Upon hyperosmotic stress, TPM4 undergoes phase separation, recruiting 10 essential glycolytic enzymes to upregulate glycolysis and support energy for actin reorganization.

We observed that TPM4 phase separation facilitated the recruitment of glycolytic enzymes onto the actin filaments, promoting glycolysis. An intriguing question arose: what are the metabolic consequences of TPM4 absence? To answer this, we generated a TPM4 knockout (KO) cell line by CRISPR/Cas9 (Supplementary Fig. S4a). In normal cells under hyperosmotic stress, there was colocalization of the PFKM and actin filaments (Pearson’s coefficient = 0.486), as evidenced by the alignment of their distribution patterns in line scanning (Fig. 5a); however, this correlation was not observed in TPM4 KO cells (Pearson’s coefficient = 0.143) (Fig. 5b). Following this, we observed that the elevation levels of lactate in the TPM4 KO group were lower compared to the control group under hyperosmotic stress (Supplementary Fig. S4b). These observations argue that TPM4 is essential for maintaining glycolytic activity under hyperosmotic stress.

In TPM4 KO cells, no significant changes were observed in actin filaments under isosmotic conditions (Supplementary Fig. S4c). However, under hyperosmotic stress, less and thin actin filaments appeared in TPM4 KO cells compared with normal cells (Fig. 5c). This finding supports that TPM4 phase separation and its recruitment of glycolytic enzymes onto actin filaments are important for actin reorganization under hyperosmotic stress.

### TPM4-PFKM condensates can be observed in physiological conditions

Many organ tissues within the body, including but not limited to renal, hepatic, and intestinal tissues, are able to maintain osmotic homeostasis. The renal tissues sustain osmotic stress as high as 1200 mOsm^54^. This raised an intriguing question of whether inherently hyperosmotic environment in renal tissues triggers TPM4 condensation. Indeed, by immunofluorescence staining we detected TPM4 condensates in mouse kidney tissues (Fig. 5d). Moreover, these condensates also displayed strong colocalization with PFKM condensates (Pearson’s coefficient = 0.78) (Fig. 5d).

In summary, our data suggest a model that actin reorganization is coupled to glycolysis through phase separation under hyperosmotic stress (Fig. 5e): the actin-binding protein TPM4 can undergo phase separation, forming condensates that wet actin filaments. TPM4 condensates are able to recruit multiple essential glycolytic enzymes including HK2, PFKM and PKM2, promoting glycolysis and subsequently facilitating actin reorganization.

## Discussion

Dynamic rearrangements of the actin cytoskeleton are critical for various cellular processes, including morphological change, cell motility, mechanical integrity and transcription regulation^55^. Studies on unstimulated platelets revealed that ~50% of total ATP consumption is dedicated to maintaining actin dynamics^1^. Similarly, in neurons, when actin turnover is inhibited, either by blocking actin disassembly or assembly, ATP depletion is reduced by 50%^2^. How does actin cytoskeleton acquire such a significant amount of ATP in time scales of rapid reorganization? Early indications that glycolysis may provide the necessary fuel for the actin cytoskeleton have emerged from studies reporting direct interactions between glycolytic enzymes and actin. For instance, phosphofructokinase-1 (PFK-1) binds to actin through electrostatic interactions in vitro^3^. ALDOA has been shown to preferentially bind to F-actin rather than G-actin in vitro^4^. Furthermore, GAPDH also directly interacts with F-actin in vitro^5^. Our observations revealed that upon exposure to hyperosmotic stress, the actin-binding protein TPM4 can undergo phase separation and recruit ten essential glycolytic enzymes. TPM4 condensates had high levels of glycolytic intermediates and ATP, suggesting that TPM4-glycolytic enzyme condensates on the actin filaments may efficiently supply energy for the dynamic actin rearrangement under hyperosmotic stress. Furthermore, exploring the energy sources and potential mechanisms of actin cytoskeleton rearrangement under other stress conditions would be a valuable avenue for future research.

Actin filaments are constantly remodeled by subunit assembly and disassembly. Endogenous TPM4 may exist in at least two populations: one population associates with the actin cytoskeleton, while the other non-actin-associated population resides in the cytoplasm^56^. Following hyperosmotic stress, we observed an increased number of barbed ends in the cells (Fig. 1b), indicating a disturbance remodeling process of the actin cytoskeleton. This process is accompanied by TPM4 phase separation. When we depolymerized the actin filaments by latrunculin B treatment, TPM4 was released from the actin cytoskeleton, concentrating in the cytoplasm and undergoing more pronounced phase separation, as revealed by the formation of more distinct condensates. When TPM4 undergoes phase separation, it forms small condensates and wets actin filaments, and these condensates are distributed throughout the cell. However, upon depolymerization of the actin filaments, these wetting condensates may lose their structural tethering from the actin and become more concentrated in the cytoplasm (Fig. 2j). Consequently, the condensates appear more prominent and evident within the cellular environment.

Normally, the ten core glycolytic enzymes are diffusely localized throughout the cytoplasm^57^. Different components could be brought together through LLPS to react efficiently^26,27^. The localization of TPM4 condensates on the actin filaments and their ability to recruit essential glycolytic enzymes, including HK2, PFKM and PKM2, suggest that TPM4-mediated glycolytic compartmentalization may enhance the efficiency of glycolysis, meeting the energy demands of actin dynamics. This observation aligns with the concept of LLPS as a platform for metabolic regulation, where the co-localization of enzymes within condensates could accelerate their activity and enhance their efficiency. When actin assembly requires a quick energy supply, actin-binding protein TPM4 undergoes phase separation and recruits glycolytic enzymes to provide energy. Similarly, when other cellular structure organization requires energy, could corresponding core proteins recruit glycolytic enzymes to supply energy? Alternatively, the ubiquitous actin cytoskeleton and its associated TPM4-glycolytic enzyme condensates may not only provide energy to actin filaments but also satisfy local cellular energy demands.

Some glycolysis-related condensates have been reported in the literature. For instance, “glucosome” is an assembly of glycolytic enzymes including PFK, FBPase, PEPCK, and PKM, which is organized to regulate glucose flux in human cancer cells^28^. Additionally, under hypoxic stress, yeast and *C. elegans* neurons form “G bodies” or “glycolytic granules” containing enzymes like PFK, potentially enhancing glycolytic activity and ATP production^30–32^. In our study, we discovered that TPM4 condensates formed through phase separation localize on the actin filaments and enrich essential glycolytic enzymes including HK2, PFKM and PKM2. TPM4-mediated glycolytic compartmentalization thus promotes glycolysis and facilitates actin reorganization, providing a novel model that links actin cytoskeleton and glycolysis. In general, these different glycolysis-related condensates may represent strategies for cells to adapt to various environmental stresses.

Limitation of this study: we did not emphasize the localized energy source, but intended to highlight the role of TPM4 phase separation in linking glycolysis and actin reorganization. While our current data suggest that TPM4 condensates might act as a local energy source for actin polymerization, we do not provide direct proof. In future studies, we aim to employ higher resolution spatiotemporal microscopy techniques coupled with compatible fluorescent probes to directly investigate whether TPM4 condensates serve as a local direct energy source for actin polymerization. Our in vivo studies on hyperosmotic stress-induced TPM4 phase separation are relatively limited, and the presence of TPM4 condensates has mainly been demonstrated through simply staining mouse kidney tissues. However, there is still a lack of substantial evidence regarding whether TPM4 in renal cortex cells and medullary cells exhibits different organizational modes and physiological functions under different osmotic environments. Further experiments are needed to observe the responses of TPM4 in different cell types within the kidney to varying osmotic stresses and determine their respective physiological functions. In our study, we focused on the spatial recruitment of various glycolysis-related enzymes by TPM4 after hyperosmotic stress but lacked the detection of enzyme activity in condensates. Exploring the activities of different enzyme components in condensates formed after phase separation is crucial for functional studies and represents a challenging and important research direction in the field of phase separation.

Overall, our findings provide novel insights into the role of TPM4 phase separation in linking glycolysis and actin reorganization, suggesting a potential mechanism for providing local energy for actin dynamics. Further investigation is needed to fully understand the functional significance of these condensates and their potential roles in other cellular processes.

## Materials and methods

### Cell lines and cell culture

Human embryonic kidney 293T (HEK293T) cells were kept by our laboratory. HeLa cells were generous gifts from Yuxin Yin laboratory (Peking University, China). MDA-MB-231 cells were generously provided by Yujie Sun laboratory (Peking University, China). Cells were cultured in Dulbecco’s modified Eagle medium (DMEM; Corning, 10-013-CRVC) supplemented with 10% fetal bovine serum (FBS; PAN-Biotech, P30-3302), 100 U/mL penicillin and 100 μg/mL streptomycin at 37 °C with 5% CO_2_. For cell passage, cells were washed with PBS (Macgene, CC010) and digested with trypsin (Macgene, CC012).

### Plasmids and transient transfection

The cells were passaged 12 h prior to plasmid transient transfection to ensure that the cell density for transfection falls within the 50%–60% range. About 1 h before transfection, the medium was replaced with fresh cell culture medium. The plasmid transient transfection system was prepared as per the instructions of Neofect (Genomtech, TF20121201). After being mixed, the plasmid transient transfection reagent was allowed to stand for 20–30 min before being evenly added to the culture dish. Around 12 h later, the medium was replaced with fresh one. The transfected plasmids in the cells exhibited high expression levels within 36–72 h, enabling live-cell imaging, immunofluorescence staining, and immunoblotting experiments.

### Antibodies and reagents

The following antibodies were used in this study: mouse anti-TPM4 (67244-1-Ig) from Proteintech, China; rabbit anti-PFKM (A5477), rabbit anti-ALDOA (A1142), rabbit anti-PKM2 (A0268), rabbit anti-HK2 (A0994), rabbit anti-GPI (A13308), rabbit anti-TPI (A15733), rabbit anti-PGK1(A14039), rabbit anti-ENO1(A1033), rabbit anti-PGAM1 (A4015) from ABclonal Technology (Wuhan, China); rabbit anti-TPM4 (EPR13316), rabbit anti-GAPDH (ab181602) from Abcam; anti-mouse (sc-516102, 1:4000) and anti-rabbit (sc-2004, 1:4000) horseradish peroxidase (HRP)-conjugated secondary antibodies from Santa Cruz Biotechnology; Alexa Fluor 488- or Alexa Fluor 555-conjugated secondary antibody from Thermo Fisher Scientific.

The following reagents were used in this study: DNA polymerase Mix (AG12210) from ACCURATE BIOTECHNOLOGY (HUNAN) CO., LTD, Changsha, China; endonuclease *Eco*RI (JE201-01), *Not*I (JN401-01), *Nco*I (JN101-01) and *Bam*HI (JB101-01) from TransGen Biotech, China; fibronectin (Sigma-Aldrich, F1056-5MG); Neofect (Genomtech, TF20121201); latrunculin B (Sigma, L5288).

### CRSPR/Cas9-mediated *TPM4* gene knockout

For *TPM4* knockout, the following sgRNAs in lentiCRISPR-V2 (#52961, Addgene) were used in MDA-MB-231 cells: 5′-cgagctggataaatattccg-3′ (sgRNA-1) and 5′-gcttctcctgcgcgtccttc-3′ (sgRNA-2). After puromycin selection, the single-cell clones were cultured and verified by sequencing and western blotting.

### Generation of TPM4-AcGFP knock-in cells

The following sgRNAs were cloned into lentiCRISPR-V2 vector. The donor templates contained left homologous arm (800 bp before the stop codon), AcGFP sequence and right homologous arm (800 bp after the stop codon). The homologous arms were amplified from the genomic DNA extracted from MDA-MB-231 cells. To generate the knock-in cells, sgRNA and relative donor template plasmid were co-transfected to the cells. After 24 h, puromycin was used to eliminate the puromycin-sensitive cells. The fluorescence-positive cells were acquired by fluorescence activated cell sorting, and the single-cell clones were cultured and verified by western blotting.

sgRNA for TPM4 C-terminal knock-in in MDA-MB-231 cells: 5′- tggtgaggattaagagtatg-3′.

### Prediction of phase separation ability

The intrinsically disordered tendency of TPM4 was predicted by MobiDB (https://mobidb.bio.unipd.it/). The residue-based *P*_DP_ of TPM4 was predicted by FuzDrop (https://fuzdrop.bio.unipd.it/predictor).

### Western blotting

For western blotting, cells were washed with PBS and lysed in an appropriate volume of RIPA buffer (50 mM Tris, pH 8.0, 150 mM NaCl, 1% Triton X-100, 0.5% Na-deoxycholate, 0.1% SDS, 1 mM EDTA and protease inhibitor cocktail) for 10 min on ice. Lysates were centrifuged at 13,572× *g* for 10 min and the supernatants were collected. Then, 5× SDS loading buffer was added to the supernatants and boiled for 10 min. Protein samples were run on 10% SDS-PAGE gels and transferred onto nitrocellulose membranes by wet electrophoretic transfer, followed by incubations with primary and secondary antibodies at 4 °C overnight or at room temperature for 2 h. A gel imaging system was used for result collection. A working solution was prepared by mixing luminescent liquid A and B at a 1:1 ratio, and then applied evenly onto the strip. After a 2-min incubation, exposure imaging was directly performed on the gel imaging instrument, and the exposure time was adjusted based on the depth of the strip.

### Proximity labeling with TurboID

MDA-MB-231 cells were seeded into a 6-cm cell culture dish, and then transient transfections of the plasmids Plvx-TPM4-Turbo, Plvx-PDLIM1-Turbo, Plvx-α-actinin1-Turbo, Plvx-ArgBP2-Turbo, and Plvx-Turbo were performed. After 24 h, the cells were passaged into a 10-cm cell culture dish, respectively. Then biotin was used for labeling at a working concentration of 50 μmol/L. The original culture medium was discarded, the prepared biotin-containing medium was added, and incubated for 10 min. After that, the culture medium was removed, and cells were washed 2–3 times with cold PBS to remove residual biotin. Protein was extracted using RIPA lysis buffer (50 mM Tris-HCl, pH 8.0, 150 mM NaCl, 1% Triton X-100, 0.5% Na-deoxycholate, 0.1% SDS, 1 mM EDTA and protease inhibitor cocktail). The streptavidin magnetic bead suspension was thoroughly mixed, 50 μL of the magnetic bead suspension was pipetted out, and a magnetic stand was used to collect beads, and then the beads were washed with lysis buffer 3 times. 1 mg of protein was taken and thoroughly mixed with the magnetic bead suspension, followed by incubation overnight at 4 °C on a shaking bed. On the following day, the overnight incubated magnetic beads were washed twice with lysis buffer, followed by washing with 1 M KCl solution, 0.1 M Na_2_CO_3_ solution, and 2 M urea (10 mM Tris-HCl, pH 8.0), and then two additional washes with RIPA lysis buffer, ensuring that each step was completed promptly. Mass spectrometry buffer (0.1% SDS in 55 mM Tris-HCl, pH 8.0) was prepared, and 6.66 mM DTT and 0.66 mM of biotin were added. 120 μL of the prepared buffer was added to the washed magnetic beads, mixed well, and boiled in a 98 °C metal bath for 10 min. 20 μL of the protein was used for immunoblotting and the remaining 100 μL of protein was stored at –80 °C for mass spectrometry identification.

### Immunofluorescence and imaging analysis

Cells were plated on acid-washed coverslips coated with 10 mg/mL fibronectin overnight. Cells were then fixed with 4% paraformaldehyde (PFA) at room temperature for 20 min, permeabilized in 0.5% Triton X-100 in PBS for 5 min, washed with PBS once for 5 min and blocked with 10% bovine serum albumin (BSA) for 1 h. Then the primary antibody was diluted 1:200 in PBS and incubated for 1 h at room temperature. After washing with PBS 3 times, the coverslips were incubated with Alexa Fluor 488- or 555-conjugated secondary antibody (1:200) for 1 h at room temperature. After another 3 washes with PBS, the coverslips were mounted with ProLong^TM^ Glass Antifade Mountant with NucBlue^TM^ Stain (P36981, Invitrogen). After mounting medium was solidified, images were captured by Andor Dragonfly confocal imaging system.

### Kidney dissection and immunofluorescence

Animal studies were conducted according to guidelines approved by the Biomedical Ethics Committee of Peking University (approval# LA2020398). Kidney tissues from two wild-type 8-week-old C57BL/6 mice were immersion-fixed with 4% PFA in PBS for 48 h at 4 °C. For the immunofluorescence staining, the kidney tissue was embedded using the frozen section embedding medium OCT and sectioned into 10-μm thick slices using a vibratome (Leica). The sections were exposed to room temperature for 2–3 h to ensure that the tissue fully adhered to the glass slide and prevent detachment. Then, the sections were fixed with 4% PFA for 30 min at room temperature. The sections were washed 2–3 times with PBS, followed by permeabilization with 0.5% Triton X-100 for 30 min at room temperature. The permeabilized sections were transferred to a humidified chamber, an immunohistochemistry pen was used to draw a circle slightly larger than the tissue around the tissue, and then the sections were blocked with 10% BSA containing 0.5% Triton X-100 at room temperature for 1 h. The sections were incubated sequentially with a primary antibody against TPM4 (1:100; Proteintech) and PFKM (1:100, Abclonal) in buffer (0.5% Triton X-100 and 2% BSA) for 2 h. After washing with PBS 3 times, the coverslips were incubated with Alexa Fluor 488-, 555-conjugated secondary antibodies (1:200) and phalloidin (1:400) for 1 h at room temperature. After another 3 washes with PBS, the coverslips were mounted with ProLong^TM^ Glass Antifade Mountant with NucBlue^TM^ Stain (P36981, Invitrogen). After mounting medium was solidified, images were captured by Andor Dragonfly confocal imaging system.

### Live-cell imaging of optoDroplet system

For long-term live-cell imaging, cells were plated on 10 mg/mL fibronectin-coated glass-bottom cell culture dishes and maintained in CO_2_-independent DMEM (Gibco, 18045-088) supplemented with 10% FBS, 100 U/mL penicillin and 100 mg/mL streptomycin at 37 °C throughout the imaging process. For optoDroplet phase separation imaging, cells were covered with tin foil to block light and images were acquired at the indicated intervals using a 63 × 1.4 NA objective lens on Andor Dragonfly confocal imaging system.

### Live-cell NADH and ATP determination

For live-cell imaging of NADH and ATP determination, cells were transfected with the NADH biosensor “Peredox-mCitrine (λ_ex_/λ_em_ = 400/510 nm)” and the ATP biosensor “MaLionR” (λ_ex_/λ_em_ = 565/585 nm). These cells were plated on 10 mg/mL fibronectin-coated glass-bottom cell culture dishes and maintained in CO_2_-independent DMEM (Gibco, 18045-088) supplemented with 10% FBS, 100 U/mL penicillin, and 100 mg/mL streptomycin at 37 °C throughout the imaging process.

### PLA

PLA was performed with Duolink kits from Sigma-Aldrich. Cells were then fixed with 4% PFA at room temperature for 20 min. After 3 times of PBS washing, the cells were permeabilized with 0.5% Triton X-100 in PBS for 5 min. Commercial blocking solution was added to the samples and incubated for 1 h at room temperature.

After blocking, the cells were incubated with the diluted antibodies for 1 h at room temperature followed by 3 times of PBS washing. The PLUS and MINUS PLA probes were mixed and diluted (1:5) in antibody diluent and incubated with samples for 30 min at 37 °C. Then, the samples were washed in 1× Wash Buffer A for 5 min twice. The ligase was diluted (1:40) in ligation buffer (1:5 diluted in H_2_O) and incubated with samples for 30 min at 37 °C, followed by washing with 1× Wash Buffer A for 2 min twice. The polymerase was diluted (1:80) in amplification stock (1:5 diluted in H_2_O) and incubated with samples for 100 min at 37 °C. The samples were then washed in 1× Wash Buffer B for 10 min twice, followed by another washing in 0.01× Wash Buffer B for 1 min. Finally, the samples were mounted with Prolong Diamond Antifade with DAPI for 30 min at room temperature. For positive control of PLA experiments, anti-tyrosinated α-tubulin and anti-α-tubulin antibodies were used to identify the positive signals. For negative control of PLA experiments, anti-PFKM or anti-GFP antibodies were used alone.

### Constructs, protein expression and purification

For recombinant protein expression in *Escherichia coli* BL21 (DE3) cells, DNA fragments were cloned into the pET28a (+) vector. Full-length TPM4-AcGFP, TPM4-RFP, PFKM-AcGFP, HK2-AcGFP, HK2-iRFP and PKM2-BFP were cloned into pET28a (+) vector with N-terminal 6× His tag. The related plasmids were transformed into *E. coli* BL21 (DE3) cells and protein expression was induced with 100 μM IPTG at 18 °C. To determine whether the protein was expressed in the inclusion body, the cells expressing proteins were lysed in PBS supplied with 1% Triton X-100 and 1 mM phenylmethanesulfonylfluoride (PMSF) using ultrasonic cell crusher and centrifuged at 13,572× *g* for 10 min. The supernatant and pellet were collected for western blotting. Then the cells expressing proteins were lysed in 1 mM Tris (2-carboxyethyl) phosphine (TCEP) and 1 mM PMSF in PBS using ultrasonic cell crusher and centrifugation at 48,380× *g* for 30 min. The supernatant was applied to a Ni-IDA beads (Smart Lifesciences) and washed with buffer containing 1 mM TCEP with 20 mM imidazole. After that, proteins were eluted with elution buffer containing 1 mM TCEP and 300 mM imidazole in PBS. Proteins were concentrated by centrifugation at 3000× *g* at 4 °C until reaching the volume of 500 μL using 10 kDa concentrator (UFC9010, Sigma-Aldrich), and then loaded onto a Superdex 200 Increase 10/300 (GE Healthcare) equilibrated with gel filtration buffer containing 20 mM Tris, pH 8.0, 500 mM NaCl, and 1 mM TCEP. Peaks containing proteins were collected and evaluated with Coomassie blue staining of SDS-PAGE gels.

### In vitro phase separation assay

The purified proteins are typically stored in a storage buffer (high-salt concentration, 500 mM NaCl, 20 mM Tris-HCl, pH 8.0), which inhibits protein phase separation. When conducting in vitro phase separation experiments, it is necessary to dilute the proteins according to the desired concentration, ensuring that the ultimately diluted proteins can undergo phase separation in a normal salt concentration phase separation buffer (150 mM NaCl, 20 mM Tris-HCl, pH 8.0, 25 mM Mg^2+^, 7.5% dextran). The prepared phase separation system is introduced into a self-made phase-separation microfluidic channel and after standing, image acquisition is performed using a 63 × 1.4 NA objective lens on Andor Dragonfly confocal imaging system.

### Detection of lactate levels

The lactate levels were measured using the Lactate Assay Kit from BioVision (ab65330/K607-100). Lactate can react with the enzymes provided in the kit, producing a noticeable absorbance at 570 nm, which can be used to assess lactate levels.

In brief, following treatment of the cells with 50 mM sorbitol or PBS for 10 min, the cells were digested, and ~2 × 10^6^ cells were suspended in DBPS, followed by two washes with PBS. Then Lactate Assay Buffer from the kit was added to lyse the cells, and the lysis was carried out on ice for 20 min with gentle tapping of the centrifuge tube to ensure thorough lysis. Following lysis, the lysate was centrifuged at 4 °C, 12,000 rpm for 10 min, and the supernatant was transferred to a new centrifuge tube for further use. The reaction system (96-well cell culture plate) was prepared according to the kit instructions, mixed well, kept away from light, and incubated at 37 °C for 30 min. Upon completion of the reaction, the absorbance at 570 nm was measured using a microplate reader.

### Seahorse assay

An XF extracellular flux analyzer (Seahorse Bioscience) was used to determine the effects of the hyperosmotic stimulation on MDA-MB-231 cells, which were seeded at 20,000 cells/well. Measurements of ECAR were performed according to the manufacturer’s instructions.

In brief, the Seahorse XF Detection System needs to be powered on in advance for at least 5 h of preheating. After seeding cells using the XF Cell Culture Microplate, 80 μL of cell suspension was added to each well (~2 × 10^4^ cells), to achieve a confluency of ~90% after cell attachment. The cell suspension was allowed to settle for 1 h before being gently placed into a 37 °C cell culture incubator. The Seahorse sensor cartridge was equilibrated with double-distilled water and then incubated overnight in the 37 °C cell culture incubator. Following this, a second equilibration was performed using preheated XF Calibrant Solution, also in the 37 °C cell culture incubator for 60–70 min.

In addition to glucose and 2-DG used in the Glycolysis Stress Test Kit, this study related to cellular energy metabolism involves assessing the impact of hyperosmotic stimulation-induced phase separation on cellular glycolysis. Therefore, sorbitol solution needs to be added during drug administration, with the control group using the Seahorse assay medium. The drug addition method involves adding a fixed volume of each drug: drug port A contains a glucose solution at a final concentration of 10 mM; drug port B contains a sorbitol solution at a final concentration of 50 mM, or the Seahorse assay medium for the control group; and drug port C contains a 2-DG solution at a final concentration of 100 mM. Upon drug addition, an equal volume of the same drug should be added to the experimental and control wells in drug ports A, B, and C. Following the addition of drugs, the system is ready for subsequent detection.

### Statistics and data display

The number of biological and technical replicates and the number of samples are indicated in figure legends, the main text and the STAR methods. The mass spectrometry data were processed using Excel (Microsoft), a final list of 28 candidate proteins was obtained by subtracting the results of the empty Turbo group from those of four groups (α-actinin1-TurboID, PDLIM1-TurboID, ArgBP2-TurboID and TPM4-TurboID), and then taking the intersection of the remaining results. Subsequent enriched pathway analysis was conducted on the Metascape website (https://metascape.org/gp/index.html#/main/step1). Seahorse’s results were analyzed and processed using the desktop version of WAVE software. Data are mean ± SEM and 95% CI as indicated in the figure legends. Student’s *t* test, two-way ANOVA analysis were performed with GraphPad Prism 8.0 and Excel. Data from image analysis were graphed using Prism 8.0.

## Supplementary information

Supplementary Figures

Supplementary Video S1

Supplementary Video S2
