# Supplementary material for: TPM4 condensates glycolytic enzymes and facilitates actin reorganization under hyperosmotic stress
Source: Cell Discov. 2024 Dec 3;10:120. doi: 10.1038/s41421-024-00744-2 (PMC11612400; doi:10.1038/s41421-024-00744-2)
Supplement: Supplementary file 1 — Supplementary Figures [file 41421_2024_744_MOESM1_ESM.pdf]

Supplementary Fig. S1: The changes in actin-binding proteins after hyperosmotic stress and Venn diagram.

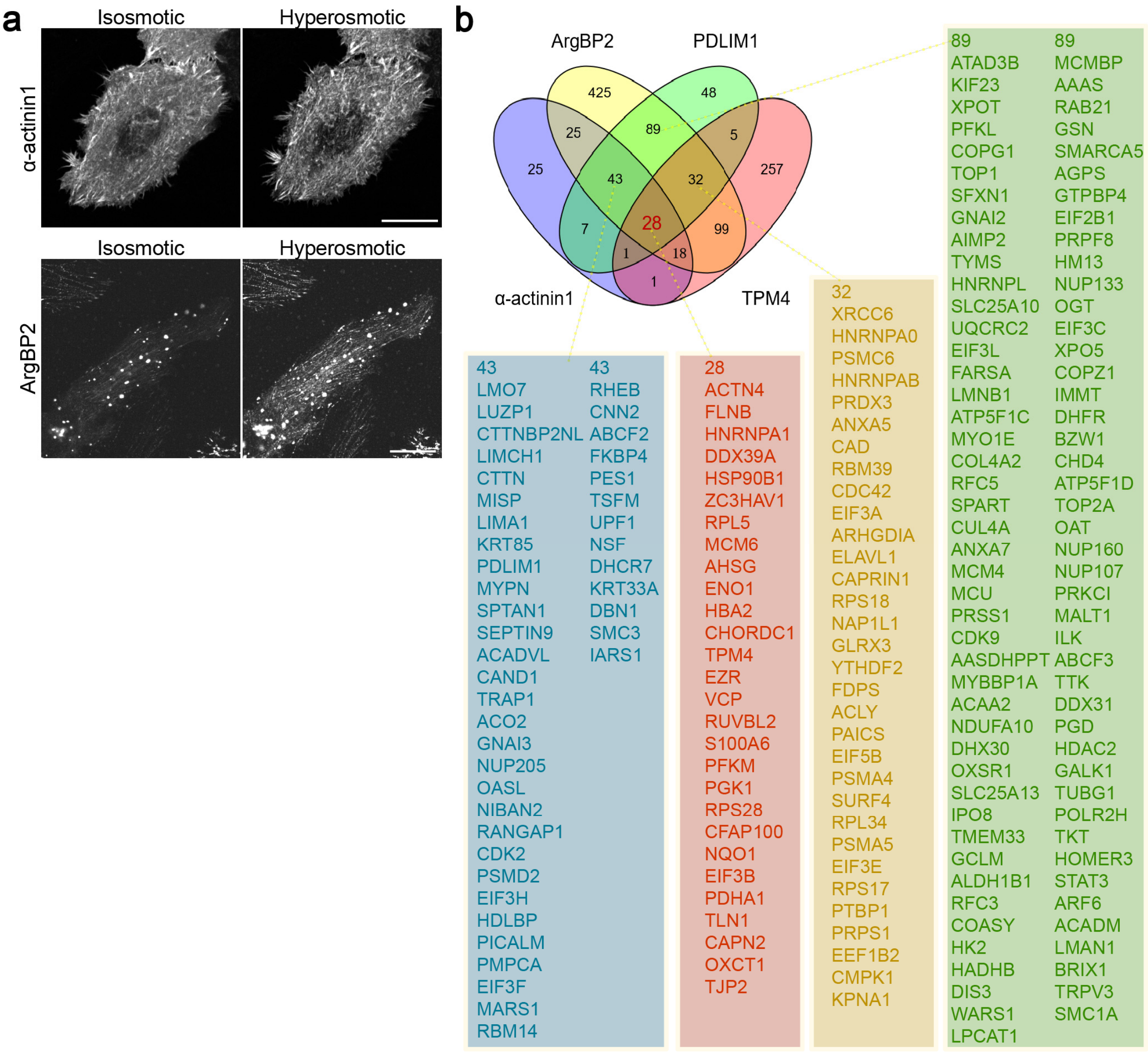

Supplementary Fig. S1: The changes in actin-binding proteins after hyperosmotic stress and Venn diagram. (a) Representative images of MDA-MB-231 cells expressing α-actinin1-AcGFP (left) or ArgBP2-AcGFP (right) before (isosmotic) and after (hyperosmotic) 100 mM sorbitol treatment for 3 min. Scale bar, 20 μm. (b) A final list of 28 candidate proteins was obtained by subtracting the results of the empty Turbo group from those of four groups (α-actinin1-TurboID, PDLIM1-TurboID, ArgBP2-TurboID and TPM4-TurboID), and then taking the intersection of the remaining results.

Supplementary Fig. S2: TPM4 condensates are dynamic and correlate with actin filaments.

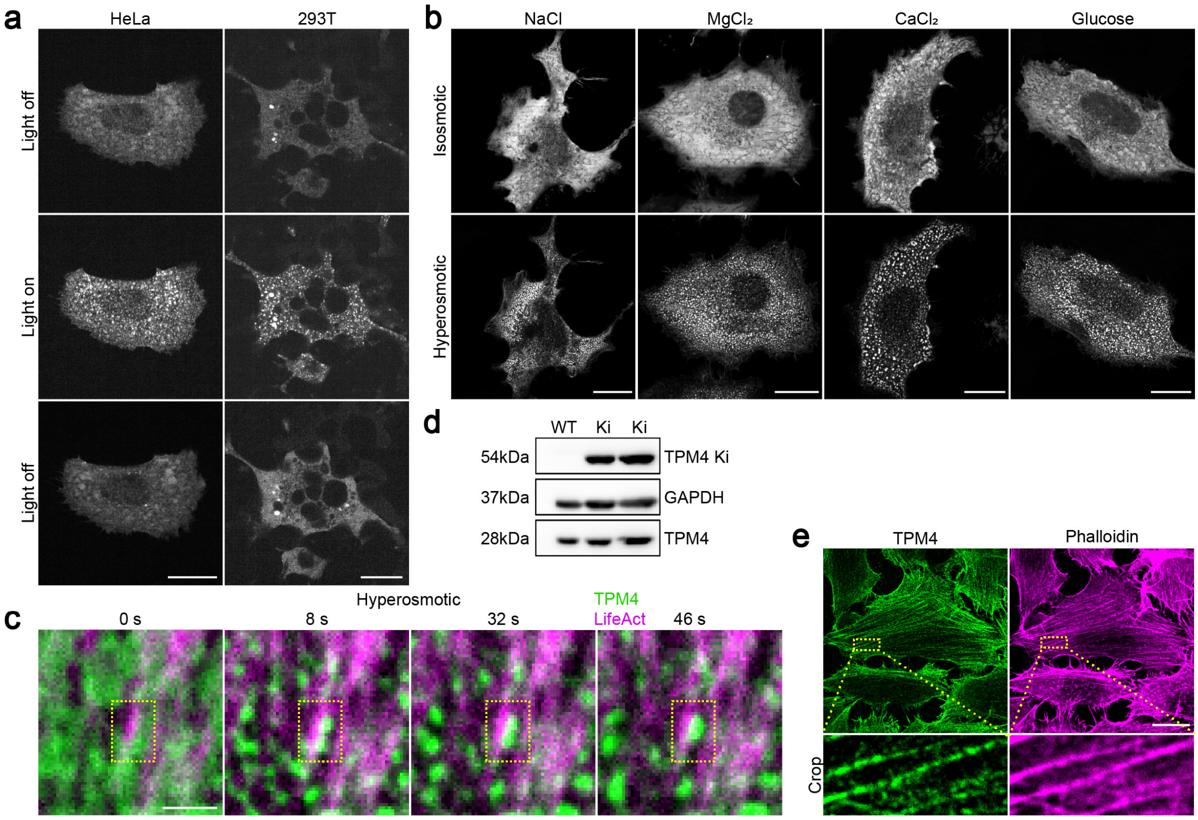

Supplementary Fig. S2: TPM4 condensates are dynamic and correlate with actin filaments. (a) Representative images of TPM4-Cry2 in HeLa and HEK 293T cells upon blue light exposure and withdrawal. Scale bar, 20 μm. (b) Representative images of TPM4-AcGFP before and after treatment with 100 mM NaCl, 100mM $MgCl_2$, 100mM $CaCl_2$ or 100 mM glucose for 3 min in MDA-MB-231 cells. Scale bar, 20 μm. (c) Representative time-lapse images of the fusion event of TPM4 condensates along actin filaments (labeled with LifeAct) in MDA-MB-231 cells treated with 100 mM sorbitol. Scale bar, 2 μm. (d) Western blot showing the successful knockin of TPM4-AcGFP in MDA-MB-231 cells. GAPDH is used as loading control. (e) Representative images of endogenous TPM4 antibody (green) and actin filaments (magenta, stained by phalloidin). Scale bar, 20 μm.

Supplementary Fig. S3: TPM4 condensates contain multiple glycolytic enzymes.

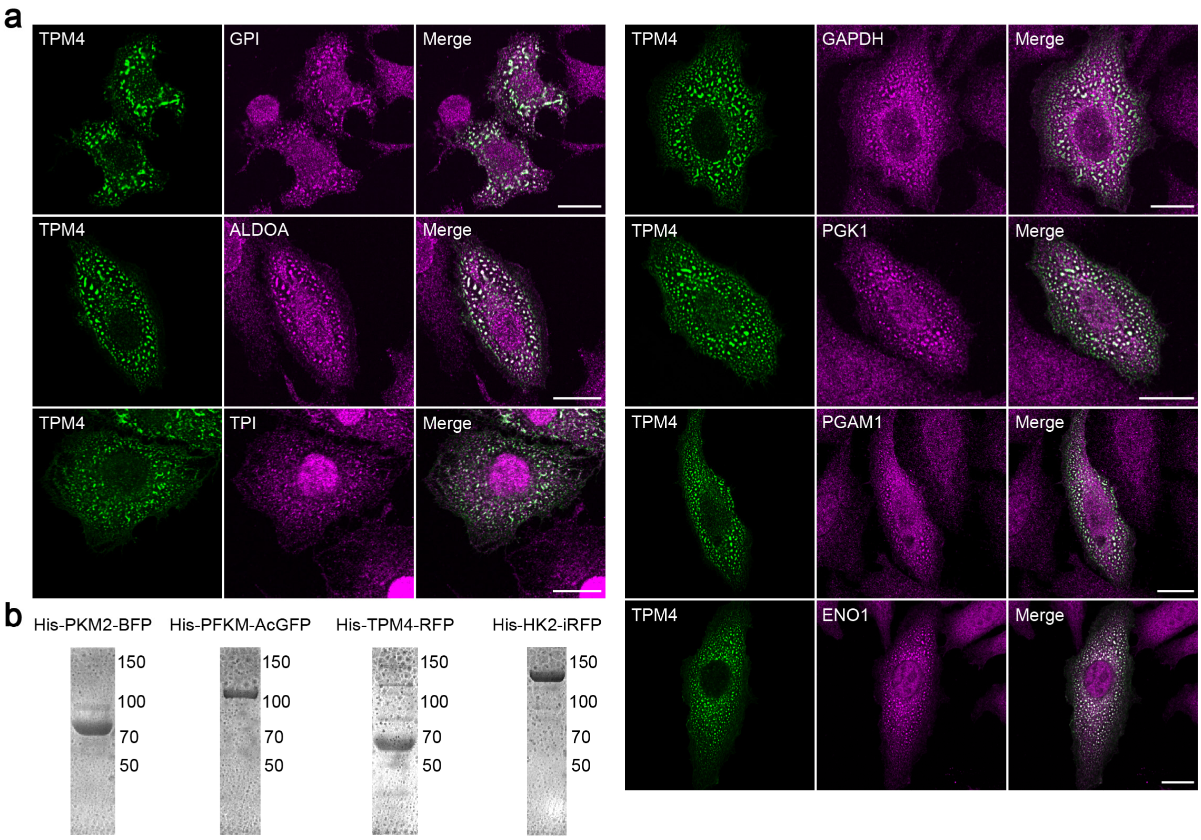

Supplementary Fig. S3: TPM4 condensates contain multiple glycolytic enzymes. (a) Representative immunofluorescence images stained with GPI, ALDOA, TPI, GAPDH, PGK1, PGAM1 and ENO1 in MDA-MB-231 cells expressing TPM4-AcGFP under hyperosmotic condition (100 mM sorbitol for 3 min). Scale bar, 20 μm. (b) Representative Coomassie-Blue-staining SDS-PAGE results of PKM2-BFP, PFKM-AcGFP, TPM4-RFP and HK2-iRFP purified from E. coli.

Supplementary Fig. S4: TPM4 KO group exhibits lower lactate levels under hyperosmotic stress, while showing no significant impact on actin filaments under isosmotic conditions.

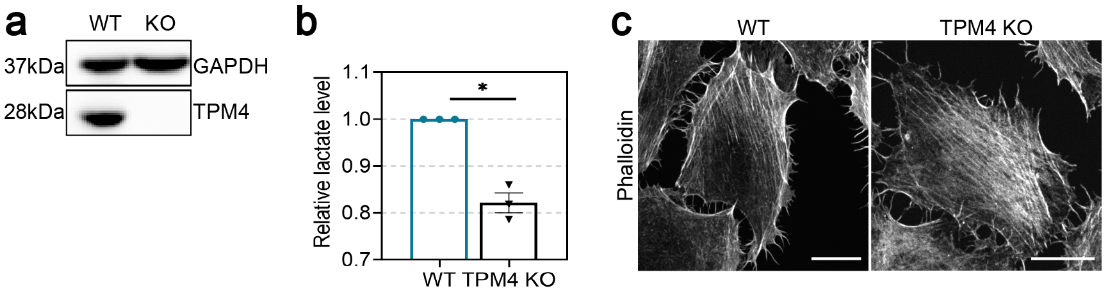

Supplementary Fig. S4: TPM4 KO group exhibits lower lactate levels under hyperosmotic stress, while showing no significant impact on actin filaments under isosmotic conditions. (a) Western blot showing the successful knockout of TPM4 in MDA-MB-231 cells. GAPDH is used as loading control. (b) Quantitation of relative lactate level in WT and TPM4 KO MDA-MB-231 cells with 100mM sorbitol treatment for 10 min (N = 3 independent experiments, error bar: mean with SEM, *p = 0.0140 by paired t test). (c) Representative immunofluorescence images stained with phalloidin in WT and TPM4 KO cells. Scale bar, 20 μm.

Supplementary Video S1: TPM4 condensates wet actin filaments under hyperosmotic stress. Representative movie of TPM4 (green) and LifeAct (magenta) in MDA-MB-231 cells treated with 100 mM sorbitol within 150 s. Scale bar, 2 μm.

Supplementary Video S2: Fusion events of TPM4 condensates occurring along actin filaments. Representative movie of TPM4 (green) and LifeAct (magenta) in MDA-MB-231 cells treated with 100 mM sorbitol within 40 s. Scale bar, 2 μm.
